# Intralesional curettage and cementation for low-grade chondrosarcoma of long bones: retrospective study and literature review

**DOI:** 10.1186/1477-7819-12-336

**Published:** 2014-11-10

**Authors:** Musa Ugur Mermerkaya, Senol Bekmez, Fatih Karaaslan, Murat Danisman, Kemal Kosemehmetoglu, Gokhan Gedikoglu, Mehmet Ayvaz, Ahmet Mazhar Tokgozoglu

**Affiliations:** Department of Orthopaedics and Traumatology, Bozok University, Yozgat, Turkey; Department of Orthopaedics and Traumatology, Dr. Sami Ulus Training and Research Hospital, Ankara, Turkey; Department of Orthopaedics and Traumatology, Hacettepe University, Ankara, Turkey; Department of Pathology, Hacettepe University, Ankara, Turkey

## Abstract

**Background:**

Various treatment strategies for low-grade chondrosarcomas with variable outcomes have been reported in the literature. The aim of this study was to assess the oncological and functional outcomes associated with intralesional curettage followed by adjuvant therapy comprising high-speed burring, thermal cauterization, and bone cementation with polymethylmethacrylate.

**Methods:**

We performed a retrospective review of 21 consecutive patients with intramedullary low-grade chondrosarcoma of long bones treated by intralesional curettage and adjuvant therapy comprising high-speed burring, thermal cauterization, and cementation at our institution from 2007 to 2012.

**Results:**

The average age of the patients was 48.7 (range, 18–71) years. There were 7 male and 14 female patients. The mean follow-up period was 58.4 (range, 26–85) months after surgery. The treated lesions were located in the proximal humerus (n =10), proximal tibia (n =6), and distal femur (n =5). At the average follow-up time point of 58.4 (range, 26–85) months, no patient had developed local recurrence and no distant metastases were observed. The average Musculoskeletal Tumor Society score among all 21 patients was 95% (84–100).

**Conclusions:**

The combination of intralesional curettage, application of high-speed burring, thermal cauterization, and cementation is an effective treatment strategy for low-grade intramedullary chondrosarcoma of long bones. Excellent oncological and functional results can be obtained.

## Background

Chondrosarcoma (CS) is the second most frequent primary malignant bone tumor after osteosarcoma. Central CS may grow primarily in the medullary canal of healthy bone or may be secondary to pre-existing benign enchondroma [1].

The prognosis of central CS is directly correlated with the histological grade of malignancy, which is assessed following the criteria described by Evans et al*.*, which consider the tumor’s cellularity, matrix characteristics, nuclear features, and mitotic rate [2]. Histologic evaluation of cartilaginous tumors represents a challenging task for the pathologist, and consistent interobserver variability in the tumor grade and distinction between benign and malignant lesions has been observed [3, 4]. The concept of the “borderline” lesion was introduced, indicating a cartilaginous lesion more active than a benign enchondroma but less atypical than a grade 1 CS [5].

The most important predictors of poor survival of patients with CS are a high histological grade and an age of more than 50 years [6]. Surgery is the primary treatment for cartilage tumors, and the extent of the resection margins depends on the tumor grade and location [7, 8]. Radiation therapy and chemotherapy have no substantial role in the treatment of CS [9–11].

Previously reported CS treatment results are difficult to interpret because of differences in grading criteria, combination with axial and appendicular tumors, and various treatment combinations [12–15]. The aim of this study is to determine the clinical outcomes of patients with grade I CS of the appendicular long bones. All patients underwent one intralesional curettage procedure followed by adjuvant therapy comprising high-speed burring, thermal cauterization, and bone cementation with polymethylmethacrylate.

## Methods

We retrospectively evaluated 21 patients treated for grade I central CS of a long bone in our hospital from 2007 to 2012 (Table 1). Patients from hospitals in the surrounding areas who were suspected of having CS were referred to our musculoskeletal oncology department.

**Table 1:** **Summary of patient data**

Patient	Sex	Age	Location	Follow-up (months)	MSTS Score (%)
1	Female	18	Proximal tibia	68	96.5
2	Female	19	Proximal tibia	39	96.5
3	Male	35	Distal femur	41	96.5
4	Female	38	Proximal humerus	48	100
5	Male	41	Distal femur	55	96.5
6	Male	41	Proximal humerus	76	96.5
7	Female	47	Distal femur	85	93.2
8	Female	47	Proximal humerus	52	100
9	Female	49	Distal femur	26	96.5
10	Female	49	Proximal humerus	80	96.5
11	Male	50	Proximal tibia	42	89.9
12	Male	53	Proximal humerus	51	100
13	Female	53	Proximal humerus	78	100
14	Female	54	Proximal humerus	55	100
15	Female	54	Proximal tibia	51	93.2
16	Female	56	Proximal humerus	60	89.9
17	Male	56	Proximal tibia	52	96.5
18	Female	58	Distal femur	65	89.9
19	Female	65	Proximal humerus	81	83.2
20	Male	69	Proximal humerus	58	93.2
21	Female	71	Proximal tibia	64	86.5

All 21 consecutive patients with histologically verified low-grade intramedullar CS of a long bone underwent intralesional curettage and cementation of their lesion at our institution. These patients were identified using our department’s histopathology database and then reviewed retrospectively. Surgery involved an oncologically safe biopsy followed by intralesional curettage, high-speed burring, and thermal cauterization. Cementation was performed immediately or 2 weeks later.

The inclusion criterion for this study was a histological diagnosis of grade I central CS located in a long bone. We excluded patients with lesions breaching the bone cortex and/or associated with a soft tissue mass because such lesions were treated by wide excision. The patients included in this study underwent intralesional curettage of their lesion through a cortical window, followed by application of high-speed burring, thermal cauterization, and bone cementation.

The resultant intraosseous defects were reconstructed with polymethylmethacrylate bone cement because it provides immediate stability, avoids morbidity of the autogenous bone graft, and aids the postoperative radiographic evaluation for signs of local recurrence [16]. Patients were admitted to the hospital for 1 to 3 days depending on the site of CS. Postoperative management was dependent upon the tumor site and bone window size. Patients with a CS in the upper extremity were managed with a sling for 2 to 6 weeks postoperatively. Following curettage in the lower extremities, patients were either non-weight-bearing or partially weight-bearing for 6 weeks and used crutches once they were mobile. None of the patients were treated with internal fixation and casts were not necessary because of the less invasive and limited nature of our surgical procedure compared with wide resection and reconstruction of the long bone.

Patients were followed by means of physical examination, radiographs, and computed tomography (CT) or magnetic resonance imaging of the extremity as well as CT of the chest (to rule out lung metastasis) every 3 months for the first 2 years, every 6 months from 2 to 5 years, and annually thereafter. Clinical outcome assessment was performed using the Musculoskeletal Tumor Society (MSTS) scoring system for the upper and lower limbs [17]. The predictive factors evaluated included radiographic evidence of endosteal scalloping, tumor size, soft tissue extension, tumor location (long bones or axial skeleton), patient age, cortical erosion on CT scans or radiographs, and the presence of preoperative pain. The outcomes included the MSTS score and tumor recurrence.

## Results

### Oncological outcome

The average age of patients was 48.7 (range, 18 to 71) years. There were 7 male and 14 female patients. The mean follow-up period was 58.4 (range, 26 to 85) months after surgery. The treated lesions were located in the proximal humerus (n =10), proximal tibia (n =6), and distal femur (n =5). At the average follow-up time point of 58.4 (range, 26 to 85) months, no patient had developed local recurrence and no distant metastases were identified.

### Histological findings

**Figure 1:**
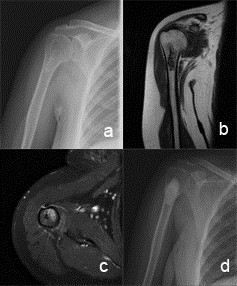
**Radiographic findings in low grade chondrosarcoma of proximal humerus. (a)** A 55-year-old female patient with a diagnosis of low-grade CS in the proximal humerus. **(b,c)** Coronaland axial magnetic resonance images at diagnosis. **(d)** Plain radiography 5 years after surgery.

**Figure 2:**
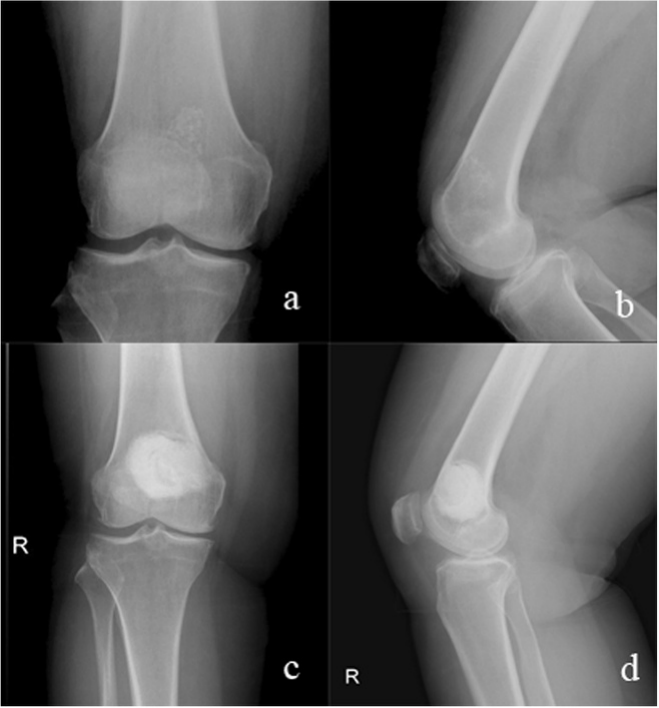
**Radiographic findings in low grade chondrosarcoma of distal femur. (a, b)** A 52-year-old female patient with a low-grade chondrosarcoma in the right distal femur. **(c**, **d)** Radiographs after 48 months from the surgery.

**Figure 3:**
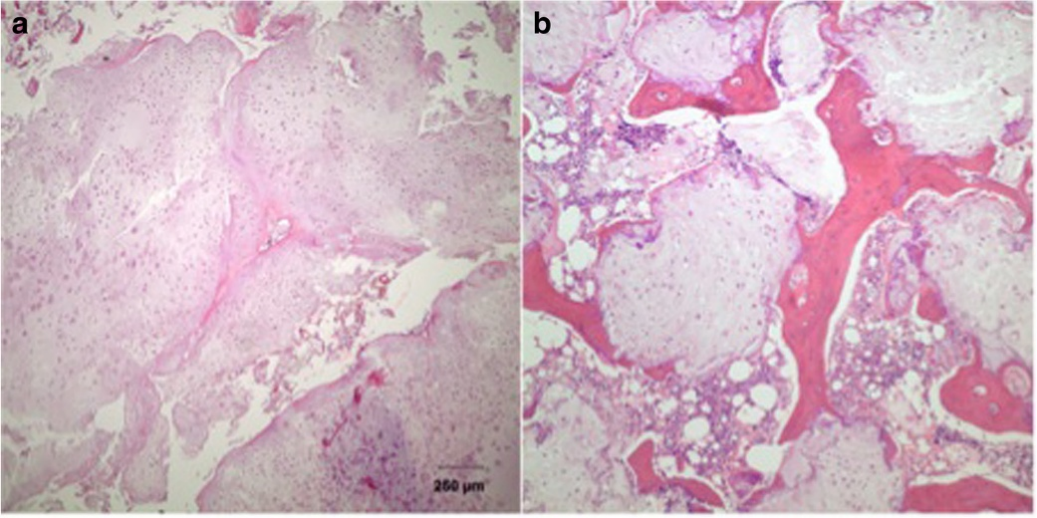
**Microscopic findings in low grade chondrosarcoma (H&E). (a)** Hypocellular chondroid lobules on low power magnification. **(b)** Host bone permeation is a hallmark of chondrosarcoma.

All patients were diagnosed with a grade I central CS according to the recently published consensus criteria [3] and the system described by Evans et al*.* [2] (Figures 1 and 2). Lobules composed of few chondrocytes within abundant chondroid matrix are consistent with chondroid neoplasm, either enchondroma or low-grade chondrosarcoma (Figure 3a); however, the presence of host bone permeation (Figure 3b) is diagnostic for low grade (grade 1) chondrosarcoma.

### Complications

One patient developed a superficial wound infection postoperatively, which was resolved with antibiotics.

### Functional outcome

The average MSTS score in all 21 patients was 95% (84% to 100%). The mean upper limb score was 96% (83% to 100%), whereas the mean lower limb score was 94% (86% to 96%). All patients were able to perform activities relating to their daily living and occupation. Two patients reported episodes of mild pain around the operation site.

## Discussion

There is currently no universally accepted operative treatment for low-grade CS of long bones. Intralesional curettage, either alone or combined with local adjuvant treatments, marginal resection, and *en bloc* resection with biologic or endoprosthetic reconstruction are described in the literature [18–20]. In contrast, high-grade CS is almost always treated with wide excision achieved by either amputation or resection of the tumor with limb salvage reconstruction [6, 12, 14, 21]. Wide resection of these indolent, slow-growing, low-grade tumors seems disproportionate in light of the difficulty in distinguishing them from enchondromas [22, 23] and the fact that wide resection often results in substantial functional morbidity [24, 25]. Hickey et al. performed a meta-analysis involving 78 patients treated with intralesional resection and 112 patients treated with wide resection for grade I CS; no significant differences in local recurrence or metastasis were found between the two methods [26]. However, the patients treated with wide resection had poorer functional outcomes. Gunay et al. performed a retrospective review of 30 consecutive patients (12 male, 18 female) with a mean age of 40.7 (range, 16 to 69) years with intramedullary low-grade CS of the long bones treated either by intralesional curettage or wide resection from 1995 to 2011 [27]. The mean overall follow-up period was 74 (range, 24 to 186) months. There was no difference in the local recurrence rate between patients treated with intralesional resection and those treated with wide resection. Intralesional curettage seems to be feasible in selected cases to reduce the patient’s postoperative morbidity in cases of grade I CS. The potential for local recurrence and metastases of low-grade CS is extremely low, with reported 5-year survival rates ranging from 85% to 100% following various treatment strategies [19].

Many authors have shown intralesional curettage to be an acceptable treatment in these cases because it avoids the morbidity associated with the more radical surgical procedures without jeopardizing the outcome [3, 28–30]. Verdegaal et al. performed a retrospective study to assess the clinical and oncological outcomes after intralesional curettage, the application of phenol and ethanol, and bone grafting in 85 patients treated from 1994 to 2005 [31]. The use of phenol as an adjuvant after intralesional curettage of low-grade CS of a long bone was safe and effective, with a recurrence rate of <6% at a mean of 6.8 years after treatment [31]. We did not use adjuvant phenol and ethanol in the present study. Residual tumors remained as a result of incomplete curettage, primarily as a consequence of a bone window that was too small or had been placed in a suboptimal location.

This study was limited by its observational and retrospective design and relatively small number of patients. We did not use a control group to compare the results. The ideal situation would be to perform a prospective, multicenter, randomized trial. Another potential limitation is the absence of low-grade CS lesions in the small tubular bones of the hands and feet in our series. However, this study supports the view that the combination of intralesional curettage, high-speed burring, and thermal cauterization is an effective treatment strategy for low-grade CS of the long bones, with excellent oncological and functional results.

## Conclusions

In conclusion, the results of intralesional curettage and cementation for the treatment of low-grade CS showed that this technique is safe and efficacious. We propose this treatment option as a rational alternative to more radical procedures because it preserves function, has low morbidity, is cost-effective, and does not appear to have an adverse effect on outcomes.

## Abbreviations

CT: Computed tomography
CS: Chondrosarcoma
MSTS: Musculoskeletal Tumor Society.
